# Enhanced Sensing and Sum-Rate Analysis in a Cognitive Radio-Based Internet of Things

**DOI:** 10.3390/s20092525

**Published:** 2020-04-29

**Authors:** Md. Sipon Miah, Kazi Mowdud Ahmed, Md. Khairul Islam, Md. Ashek Raihan Mahmud, Md. Mahbubur Rahman, Heejung Yu

**Affiliations:** 1 Department of Information and Communication Technology (ICT), Islamic University, Kushtia 7003, Bangladesh; mowdudice08@gmail.com (K.M.A.); ashekraihan.iubd@gmail.com (M.A.R.M.); mrahman@ice.iu.ac.bd (M.M.R.); 2 Department of Biomedical Engineering (BME), Islamic University, Kushtia 7003, Bangladesh; khairul.ice06@gmail.com; 3 Department of Electronics and Information Engineering, Korea University, Sejong 30019, Korea

**Keywords:** cognitive radio, Internet of Things, Kullback–Leibler divergence, energy detection, spectrum sensing, sum rate

## Abstract

Spectrum sensing plays a vital role in cognitive radio networks (CRNs) for identifying the spectrum hole. However, an individual cognitive radio user in a CRN does not obtain sufficient sensing performance and sum rate of the primary and secondary links to support the future Internet of Things (IoT) using conventional detection techniques such as the energy detection (ED) technique in a noise-uncertain environment. In an environment comprising noise uncertainty, the performance of conventional energy detection techniques is significantly degraded owing to the noise fluctuation caused by the noise temperature, interference, and filtering. To mitigate this problem, we present a cooperative spectrum sensing technique that comprises the use of the Kullback–Leibler divergence (KLD) in cognitive radio-based IoT (CR-IoT). In the proposed method, each unlicensed IoT device that is capable of spectrum sensing, which is called a CR-IoT user, makes a local decision using the KLD technique. The spectrum sensing performed with the KLD requires a smaller number of samples than other conventional approaches, e.g., energy detection, for reliable sensing even in a noise uncertain environment. After the local decision is made, each CR-IoT user sends its own local decision result to the corresponding fusion center, which makes a global decision using the soft fusion rule. The results obtained through simulations show that the proposed KLD scheme achieves a better sensing performance, i.e., higher detection and lower false-alarm probabilities, enhances the sum rate, and reduces the total time as compared to the conventional ED scheme under various fading channels.

## 1. Introduction

Internet of Things (IoT) can be regarded as one of most promising network applications that enable communications among sensor nodes, a continuous data exchange between a source and destination, and the ability to join and leave the network spontaneously [1,2,3,4,5,6]. However, the great challenges of the future IoT, which may be induced by supporting a large number of devices and various applications requiring greater bandwidth, are the spectrum scarcity problem, high implementation cost, and higher energy consumption than the general radio platforms [7,8,9,10].

Cognitive radio (CR) is a revolutionary wireless technology that enhances spectrum use by utilizing the spectrum flexibly, intelligently, and effectively [11,12,13,14,15,16]. In a cognitive radio-based IoT (CR-IoT) network [4,17,18], each secondary CR-IoT user, that uses the spectrum only when the spectrum is vacant, adopts the spectrum sensing capability. By sensing the spectrum, CR-IoT users discover the spectrum holes and choose the channel that is most suitable for their secondary communications. Owing to the precondition of channel access for CR-IoT users, each user accesses the licensed spectrum opportunistically without any harmful interference to the licensed primary user (PU). Upon returning the PU, the CR-IoT user relinquishes the spectrum because it is licensed not by CR-IoT users but by the PU.

Methods of spectrum sensing can be categorized into several types, including non-coherent detection, coherent detection, non-cooperative detection, and cooperative detection. In a non-coherent detection method, spectrum sensing is accomplished without need for any prior knowledge of the PU’s signal. In contrast, prior knowledge of the PU’s signal—e.g., training and pilot patterns—is actually required in a coherent detection scheme. As another criterion of classification, cooperation among CR-IoT users is considered. In non-cooperative detection, the detection of the PU’s signal is based on the local observation of a single CR-IoT user. In this case, therefore, the efficiency of spectrum sensing is degraded owing to a hidden terminal problem, multi-path fading, and shadow effects [19]. In cooperative detection approaches [20,21], where multiple CR-IoT users cooperatively perform spectrum sensing, can resolve the problem of local sensing. In cooperative sensing, each individual CR-IoT user performs local sensing independently and then forwards the sensing result to the fusion center (FC) through the noise-free or noisy reporting channel between the CR-IoT users and the FC. With these reported local results, the FC makes a global decision based on a fusion rule [22,23,24].

Spectrum-sensing techniques, like energy detection (ED), matched filter detection, cyclostationary feature detection, entropy-based detection, and eigenvalue-based detection, were investigated under varying conditions [25,26,27,28,29,30]. Among the previously mentioned techniques of spectrum sensing, the ED technique has the advantages of low complexity and cost effectiveness. Hence, this ED approach is especially appropriate for performing spectrum sensing without any prior knowledge about the signal pattern of the PU. Therefore, it is broadly accepted as one of the most commonly used techniques for spectrum sensing in various sensing and detection applications. Moreover, the ED technique is a blind process that does not require information about wireless channel gains and other parameter estimates about the PU’s signal. However, the exact information of the noise power at the receiver side, i.e., the sensing side, of the CR-IoT user is essential for an accurate detection. In an environment comprising noise uncertainty [31], the performance degradation of the ED technique is inevitable. Even in the cooperative sensing approach, the performance gain obtained with cooperation can be limited [32]. For the low-power operation of the CR-IoT users, a sensing time, i.e., the number of signal samples for the spectrum sensing, should be minimized as much as possible. With such a short sensing interval, the conventional ED technique is not suitable for the detection of the PU’s signal.

To overcome the problem in spectrum sensing for CR-IoT networks, a cooperative sensing mechanism was investigated. In [33], the performance limits of cooperative spectrum sensing were studied under a scenario where malicious users send false sensing data to an FC, i.e., with Byzantine attacks. In [34], the authors summarized blind spectrum sensing approaches for an interweave cognitive radio network model. It provided background, implementation, and limitation of the blind spectrum sensing approaches, such as an ED, maximum to minimum eigenvalue, maximum eigenvalue, covariance absolute value, and covariance norm approaches. In [35], the authors investigated on reliable transmission of local sensing results which are transmitted via the reporting channel to an FC from secondary users. In [36], CR-IoT users perform their local sensing, report soft energies to the FC, and store this information in their local database. The FC determines the Kullback–Leibler divergence (KLD) score against each CR-IoT user and also provides this same information to the CR-IoT user. A normally declared user attempts to send the mean of the previous energy reports to the FC based on its current observation. In [36,37], the authors used the KLD technique to evaluate the dissimilarity in the probability distribution functions under the presence and absence hypotheses of the PU’s signal. In [38], each CR-IoT user provided an FC with information about their local spectrum observations of the licensed spectrum. The FC collected the local sensing results and made its global decision. Before making any global decision, the FC assigns weights to the local sensing information of the CR-IoT users. The weights are proportional to the reliability of the local-spectrum-sensing information. However, the existing spectrum sensing performed based on the KLD techniques was evaluated in fading channels.

The main contributions of this paper can be summarized as follows:

• We propose an enhanced spectrum sensing mechanism. The sum rate in a CR-IoT networks realized using the KLD technique is evaluated and investigated. The effectiveness of the proposed scheme is verified by comparing the numerical performance, e.g., sensing performance and throughput, with the conventional ED technique.
• We review the KLD technique in which each CR-IoT user achieves the desired sensing performance, even with a small number of samples, and robustness to noise uncertainty.
• We study the sensing performance of CR-IoT users and an FC using the soft fusion rule.
• Based on the improved sensing performance, the sum rate of the primary and secondary networks, i.e., the CR-IoT, is analyzed for the conventional and the proposed scheme using the soft fusion rule under various channel conditions.

The remainder of this paper is organized as follows. Section 2 describes the system model and ED technique. Section 3 includes the proposed scheme for the future IoT wherein the analyses of both the spectrum sensing and sum rate are performed based on the KLD technique under different channels. The simulation results and discussion are presented in Section 4. We compare our proposed scheme with other existing schemes to demonstrate the sensing performance gain, enhanced sum rate, and reduced total time. Finally, our conclusion and potential future works are presented in Section 5.

## 2. Spectrum Sensing in CR-IoT Networks

Spectrum sensing is a basic and essential mechanism for CR networks to find the unused spectrum that is allocated for the PUs. In this section, an overview of the proposed system model and the conventional ED technique are presented.

### 2.1. System Model

**Figure 1:**
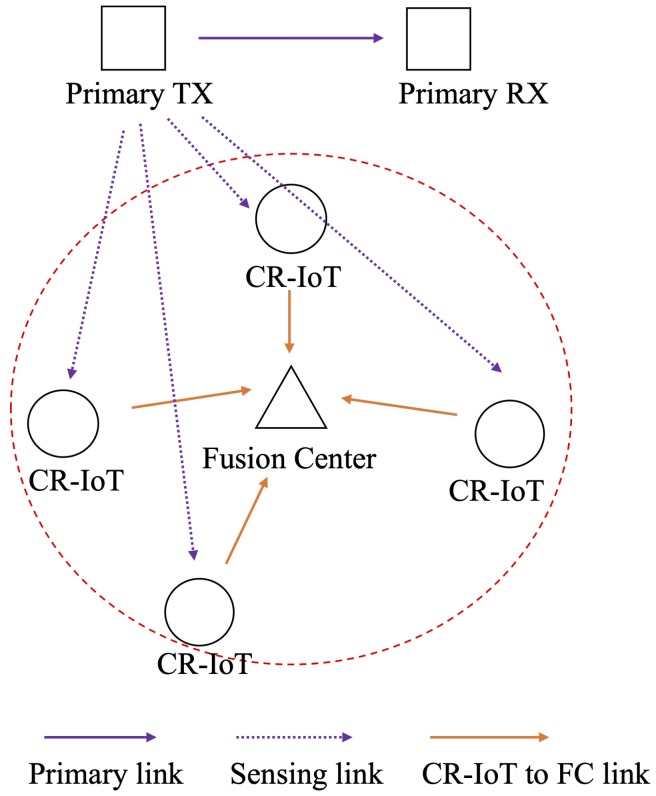
Proposed system model with a primary link and a cognitive radio-based Internet of Things (CR-IoT) network including a fusion center (FC).

The proposed system model consists of a primary link and a CR-IoT network, as shown in Figure 1. The primary link consists of the primary transmitter and receiver. The operation of the PU is considered to be a time division multiplexing access. In contrast, the CR-IoT network consists of *M* unlicensed CR-IoT users and an FC. Although each CR-IoT user has their counterpart, i.e., the corresponding receiver, the receivers are omitted from Figure 1.

In a binary hypothesis testing problem, we define the hypotheses representing the absence and presence of the PU’s signal as follows:

(1) $$\left\{\begin{array}{cc} H_{0}: & \mathrm{if}\;\mathrm{the}\;\mathrm{PU}\prime s\;\mathrm{signal}\;\mathrm{is}\;\mathrm{absent},\\ H_{1}: & \mathrm{if}\;\mathrm{the}\;\mathrm{PU}\prime s\;\mathrm{signal}\;\mathrm{is}\;\mathrm{present}. \end{array}\right.$$

Depending on the PU’s transmission, the received signal of the $i^{th}$ CR-IoT user in both binary hypotheses can be expressed as follows [39]:

(2) $$z_{i}\left(n\right)=\left\{\begin{array}{cc} y_{i}\left(n\right) & :H_{0}\\ h_{i}\left(n\right)x\left(n\right)+y_{i}\left(n\right) & :H_{1} \end{array}\right.$$

where $z_{i}\left(n\right)$ denotes the signal received by the $i^{th}$ CR-IoT user in the $n^{th}$ sample time, and $h_{i}\left(n\right)$ is the channel gain between the $i^{th}$ CR-IoT user and the primary transmitter for i=1,2,⋯,M and $n=1,2,\cdots ,N_{s}$. It is assumed that the channel is static during each sensing period. Moreover, $x\left(n\right)$ is a signal transmitted from the PU, which is modulated by a binary phase shift keying (BPSK) with a power of $p_{x}^{2},$, and $y_{i}\left(n\right)$ is a circularly symmetric complex Gaussian noise at the $i^{th}$ CR-IoT user with a variance of $\sigma_{y,i}^{2}$.

### 2.2. Conventional Energy Detection Technique

In the ED method, the received signal energy for a given time period is measured and compared with the threshold [13,14]. For each CR-IoT user to obtain the decision statistics for the ED, the time-domain signal power occupying a particular frequency band is measured as follows. First, the received signal is passed through a band-pass filter to select the appropriate signal bandwidth, and the output of this filter is then transformed by an analog-to-digital converter (ADC). Here, the analog signal is sampled to obtain a discrete signal, which is individually averaged and squared for the conventional ED technique to estimate its own received signal energy [40]. The measured energy at the $i^{th}$ CR-IoT user is expressed as follows.

(3) $$e_{i}=\sum_{n=1}^{N_{s}}{\left|z_{i}\left(\frac{n}{f_{s}}\right)\right|}^{2},$$

where $z_{i}\left(\frac{n}{f_{s}}\right)$ is the $n^{th}$ sample of a signal received by the $i^{th}$ CR-IoT user, and $N_{s}$ denotes the total number of signal samples used for sensing with a sampling frequency of $f_{s}$. Therefore, the duration of the sensing time slot is given by $\tau_{s}=N_{s}f_{s}$, which is commonly used by all CR-IoT users in a CR-IoT network.

### 2.3. Sensing Performance

Based on the center limit theorem, the distribution of the decision statistic for the $i^{th}$ CR IoT user $e_{i}$ under both hypotheses can be expressed as

(4) $$e_{i}∼\left\{\begin{array}{c} ℵ\left(\mu_{0,i}\left(H_{0}\right),\sigma_{0,i}^{2}\left(H_{0}\right)\right)\\ ℵ\left(\mu_{1,i}\left(H_{1}\right),\sigma_{1,i}^{2}\left(H_{1}\right)\right) \end{array}\right.$$

where

$$\begin{array}{cc} \mu_{0,i}\left(H_{0}\right) & =N_{s}\sigma_{z,i}^{2},\\ \sigma_{0,i}^{2}\left(H_{0}\right) & =N_{s}\sigma_{z,i}^{4},\\ \mu_{1,i}\left(H_{1}\right) & =N_{s}\left(1+|h_{i}{|}^{2}\gamma_{i}\right)\sigma_{z,i}^{2},\\ \sigma_{1,i}^{2}\left(H_{1}\right) & =N_{s}\left(1+2|h_{i}{|}^{2}\gamma_{i}\right)\sigma_{z,i}^{4}, \end{array}$$

and $\gamma_{i}$ is a signal-to-noise ratio (SNR) that is defined as $\gamma_{i}=\frac{p_{x}^{2}}{\sigma_{y,i}^{2}}$.

Based on Equation (4), we can calculate the probability of a false alarm $p_{f,i}$ and the probability of detection $p_{d,i}$ for the $i^{th}$ CR-IoT user by comparing $e_{i}$ with a pre-defined local threshold $\lambda_{i}^{ED}$ as follows.

(5) $$\begin{array}{c} p_{f,i}=Pr\left[e_{i}\geq \lambda_{i}^{ED}|H_{0}\right]=Q\left(\frac{\lambda_{i}^{ED}-\mu_{0,i}\left(H_{0}\right)}{\sigma_{0,i}\left(H_{0}\right)}\right)=Q\left(\frac{\lambda_{i}^{ED}}{\sqrt{N_{s}}\sigma_{x,i}^{2}}-\sqrt{N_{s}}\right), \end{array}$$

and

(6) $$\begin{array}{c} p_{d,i}=Pr\left[e_{i}\geq \lambda_{i}^{ED}|H_{1}\right]=Q\left(\frac{\lambda_{i}^{ED}-\mu_{1,i}\left(H_{1}\right)}{\sigma_{1,i}\left(H_{1}\right)}\right)=Q\left(\frac{\lambda_{i}^{ED}}{\sqrt{N_{s}\left(1+2\right|h_{i}{|}^{2}\gamma_{i})}\sigma_{x,i}^{2}}-\frac{\sqrt{N_{s}}\left(1+\right|h_{i}{|}^{2}\gamma_{i})}{\sqrt{\left(1+2|h_{i}{|}^{2}\gamma_{i}\right)}}\right), \end{array}$$

where $Q\left(x\right)$ denotes a Gaussian tail function that is defined as $Q\left(x\right)=\frac{1}{\sqrt{2\pi }}\int_{x}^{\infty }e^{-\frac{t^{2}}{2}}dt$.

The probability of a false alarm $p_{f,i}$ is the probability that the CR-IoT user incorrectly declares that the PU exists although the PU is actually absent. In contrast, the probability of detection $p_{d,i}$ denotes the probability that the CR-IoT user correctly declares that the PU is present.

At the FC, all the CR-IoT users send their local decisions to the FC, which are combined with the local results to obtain a global decision about the PU’s occupancy of the spectrum [39]. The sensing performance, i.e., $\left(p_{f,FC}^{ED}/p_{d,FC}^{ED}\right)$, of the global decision is given by

(7) $$p_{f,FC}^{ED}=\left\{\begin{array}{cc} 1, & if\sum_{i=1}^{M}p_{f,i}<\beta_{ED},\\ 0, & otherwise, \end{array}\right.$$

and

(8) $$p_{d,FC}^{ED}=\left\{\begin{array}{cc} 1, & if\sum_{i=1}^{M}p_{d,i}\geq \beta_{ED},\\ 0, & otherwise, \end{array}\right.$$

where $\beta_{ED}$ denotes the global decision threshold at the FC.

We can now calculate the decision statistics using Algorithm 1. Here, each CR-IoT user measures the received signal energy with $N_{s}$ samples. The locally measured energy is reported to the FC. Finally, the FC computes the global detection $\left(p_{f,FC}^{ED}/p_{d,FC}^{ED}\right)$. Based on the global sensing performance $\left(p_{f,FC}^{ED}/p_{d,FC}^{ED}\right)$, the sum rate $R^{ED}$ can be evaluated.

(9) $$R^{ED}=\alpha p_{d,FC}^{ED}R_{PU}+\left(1-\alpha \right)\left(1-p_{f,FC}^{ED}\right)R_{CR-IoT},$$

where α, $R_{PU}$, and $R_{CR-IoT}$ are defined in Section 3.2.
**Algorithm 1** The conventional ED technique with cooperation for a CR-IoT network**Input: $M,N_{s},f_{s},T,\tau_{s},$ and $\tau_{r}$****Output: Calculate the probability of a false alarm $p_{f,FC}^{ED},$ the probability of detection $p_{d,FC}^{ED},$ and the sum rate $R^{ED}$**1:Initialize $N_{s},M$  2:**for**i=1 to *M* **do**3:Set: $p_{f,i}=Pr\left[e_{i}\geq \lambda_{i}^{ED}|H_{0}\right]$  4:Set: $p_{d,i}=Pr\left[e_{i}\geq \lambda_{i}^{ED}|H_{1}\right]$  5:**end for** 6:Calculate: $p_{f,FC}^{ED}=\left\{\begin{array}{ll} 1; & if\sum_{i=1}^{M}p_{f,i}<\beta_{ED}\\ 0; & otherwise \end{array}\right.$  7:Calculate: $p_{d,FC}^{ED}=\left\{\begin{array}{ll} 1; & if\sum_{i=1}^{M}p_{d,i}\geq \beta_{ED}\\ 0; & otherwise \end{array}\right.$  8:Calculate: $R^{ED}=\alpha p_{d,FC}^{ED}R_{IoT}+\left(1-\alpha \right)\left(1-p_{f,FC}^{ED}\right)R_{CR-IoT}$

## 3. Proposed Spectrum Sensing

For the proposed spectrum sensing in a CR-IoT network, the sensing based on the KLD technique is discussed, and the sum rate analysis is provided.

### 3.1. Spectrum Sensing Based on the KLD Technique

**Figure 2:**
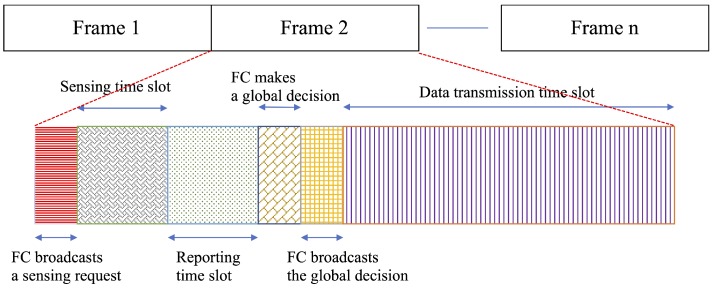
Frame structure of a time slot for reporting sensing information and packet transmission [39].

Under the frame structure presented in Figure 2, all the CR-IoT users sense the PU’s channel during a sensing time slot $\tau_{s}$ using the KLD technique.

The KLD, which is the relative entropy between two probability density functions (PDF) g(y) and f(y), is defined as [36,37,38]

(10) $$KLD\left(g||f\right)=\int g\left(y\right)\times log\left(\frac{g(y)}{f(y)}\right)dy.$$

The KLD representation of the two Gaussian distributions of $e_{i}\left(H_{1}\right)$ and $e_{i}\left(H_{0}\right)$ in Equation (4) with $H_{1}$ and $H_{0}$, respectively, is required to be evaluated. To this end, means are calculated under two hypotheses with update equations as follows:

(11) $$\begin{array}{cc} \mu_{0,i} & ={\tilde{\mu }}_{0,i}+e_{i}\left(H_{0}\right),\\ \mu_{1,i} & ={\tilde{\mu }}_{1,i}+e_{i}\left(H_{1}\right), \end{array}$$

where $\mu_{0,i}$ and $\mu_{1,i}$ are the updated mean values for the $i^{th}$ CR-IoT user and are updated with the previous mean values ${\tilde{\mu }}_{0,i}$ and ${\tilde{\mu }}_{1,i}$ and the received energy $e_{i}$ under the hypotheses.

In the same manner, the variances are updated based on the received energy $e_{i}$ under two hypotheses as follows:

(12) $$\begin{array}{cc} \sigma_{0,i}^{2} & ={\tilde{\sigma }}_{0,i}^{2}+{\left[e_{i}\left(H_{0}\right)-\mu_{0,i}\right]}^{2},\\ \sigma_{1,i}^{2} & ={\tilde{\sigma }}_{1,i}^{2}+{\left[e_{i}\left(H_{1}\right)-\mu_{1,i}\right]}^{2}, \end{array}$$

where $\sigma_{0,i}^{2}$ and $\sigma_{1,i}^{2}$ are the updated variance values for the $i^{th}$ CR-IoT user and are updated with the previous variance values ${\tilde{\sigma }}_{0,i}^{2}$ and ${\tilde{\sigma }}_{1,i}^{2}$ under two different hypotheses.

After updating the mean and variance information on behalf of all *M* CR-IoT users, the particular CR-IoT user measures the difference in the mean and variance of the $i^{th}$ CR-IoT user energy statistics from those of all other CR-IoT users. For all *M* CR-IoT users, the average values of mean are measured with the new mean values of Equation (11) and variance values of Equation (12). We can then calculate the average means, ${\bar{\mu }}_{0,i}$ and ${\bar{\mu }}_{1,i}$, as follows:

(13) $$\begin{array}{cc} {\bar{\mu }}_{0,i} & =\frac{\sum_{i=1}^{M}\mu_{0,i}-\mu_{0,i}}{\left(M-1\right)},\\ {\bar{\mu }}_{1,i} & =\frac{\sum_{i=1}^{M}\mu_{1,i}-\mu_{1,i}}{\left(M-1\right)}, \end{array}$$

where ${\bar{\mu }}_{0,i}$ and ${\bar{\mu }}_{1,i}$, under the hypotheses, are the average mean values of the energy measurements which are provided by all other users except the $i^{th}$ CR-IoT user.

Furthermore, we can calculate the average variances ${\bar{\sigma }}_{0,i}^{2}$ and ${\bar{\sigma }}_{1,i}^{2}$ as follows:

(14) $$\begin{array}{cc} {\bar{\sigma }}_{0,i}^{2} & =\frac{\sum_{i=1}^{M}\sigma_{0,i}^{2}-\sigma_{0,i}^{2}}{\left(M-1\right)},\\ {\bar{\sigma }}_{1,i}^{2} & =\frac{\sum_{i=1}^{M}\sigma_{1,i}^{2}-\sigma_{1,i}^{2}}{\left(M-1\right)}, \end{array}$$

where the average variances ${\bar{\sigma }}_{0,i}^{2}$ and ${\bar{\sigma }}_{1,i}^{2}$ are values of the energy measurements which are provided by all other users while ignoring the variance of the $i^{th}$ CR-IoT user. That is, these variances are obtained by excluding the $i^{th}$ CR-IoT user.

With Equations (13) and (14), we obtain *M* different statistics for the channel sensing with different combinations of CR-IoT users. Under the fading channels, some of the CR-IoT users could experience significantly deep fading. In this case, these CR-IoT users may report incorrect sensing results owing to the channel fading effects. To mitigate such a problem, we employ multiple decision statistics based on the various combinations of CR-IoT users. Using this approach, we can prevent the occurrence of a global channel sensing error caused by a few users under deep-fading channels.

After calculating the means and variances, the $i^{th}$ CR-IoT user obtains a local decision-statistics- based KLD as follows:

(15) $$\begin{array}{cc} \; & KLD\left(i\right)=KLD\left({\bar{\mu }}_{0,i},{\bar{\mu }}_{1,i},{\bar{\sigma }}_{0,i}^{2},{\bar{\sigma }}_{1,i}^{2}\right)\\ \; & \;\;=\frac{1}{2}\left[\log\left(\frac{{\bar{\sigma }}_{0,i}^{2}}{{\bar{\sigma }}_{1,i}^{2}}\right)-1+\left(\frac{{\bar{\sigma }}_{1,i}^{2}}{{\bar{\sigma }}_{0,i}^{2}}\right)+\frac{{\left({\bar{\mu }}_{1,i}-{\bar{\mu }}_{0,i}\right)}^{2}}{{\bar{\sigma }}_{0,i}^{2}}\right]. \end{array}$$

All the cooperative CR-IoT users provide the information of their local decision statistics to the FC. The FC then collects the information and makes a global decision. We can then evaluate the sensing performance $\left(p_{f,FC}^{KLD}/p_{d,FC}^{KLD}\right)$ based on the KLD statistics of the reporting CR-IoT users as follows:

(16) $$p_{f,FC}^{KLD}=\left\{\begin{array}{cc} 1, & if\sum_{i=1}^{M}KLD\left(i\right)<\beta_{KLD},\\ 0, & otherwise, \end{array}\right.$$

and

(17) $$p_{d,FC}^{KLD}=\left\{\begin{array}{cc} 1, & if\sum_{i=1}^{M}KLD\left(i\right)\geq \beta_{KLD},\\ 0, & otherwise. \end{array}\right.$$

where $\beta_{KLD}$ denotes the global decision threshold at the FC.

In the proposed sensing scheme, the probabilities of false alarm and detection cannot be analyzed because the distribution of the KLD is not available. For the evaluation of the sensing performance, therefore, the numerical results should be employed.

### 3.2. Sum Rate Analysis

Using the frame structure and sensing performance in the above subsection, we can analyze the sum rate while using several assumptions. In the transmission slot, the CR-IoT transmitter sends data according to scheduling based on a round-robin manner.

In a non-false alarm event, i.e., the absence of the PU is accurately detected by the unlicensed CR-IoT when the PU is absent, the unlicensed CR-IoT user can access the primary spectrum with the probability $\left(1-p_{f,FC}^{KLD}\right)$. In a detection event, the PU’s transmission is not interfered by the CR-IoT users. Therefore, the sum rate of both the PU and the CR-IoT users with a round-robin scheduling is expressed as

(18) $$R^{KLD}=\alpha p_{d,FC}^{KLD}R_{PU}+\left(1-\alpha \right)\left(1-p_{f,FC}^{KLD}\right)R_{CR-IoT},$$

where $R_{PU}$ denotes the channel capacity of the PU link, $R_{CR-IoT}$ is the channel capacity of the CR-IoT link, and $\alpha \in \left[0,1\right]$ denotes the primary activity factor, which indicates the probability of the PU’s transmission in a given frame. $R_{PU}$ and $R_{CR-IoT}$ are defined as follows:

(19) $$R_{PU}=\log_{2}\left(1+SNR_{PU}\right),$$

and

(20) $$R_{CR-IoT}=\frac{T-\tau_{s}}{T}\sum_{i=1}^{M}\log_{2}\left(1+SNR_{CR-IoT,i}\right),$$

where $SNR_{PU}$ and $SNR_{CR-IoT,i}$ denote the SNR of the PU’s link and the $i^{th}$ CR-IoT link, respectively, and *T* denotes the total frame length.

In Algorithm 2, the entire process for obtaining the global decision at the FC and the sum rate evaluation are described.

### 3.3. Total Time Analysis

In the proposed scheme, if we increase the number of CR-IoT users (*M*), the total sensing and reporting time $\tau_{t}$, consisting of the sensing time $\tau_{s}$ and reporting time $\tau_{r}$, will be increased [34]. The total time required by the proposed scheme can be calculated as follows:

(21) $$\tau_{t}=\tau_{s}+\sum_{i=1}^{M}\tau_{r,i}=\tau_{s}+\sum_{i=1}^{M}\tau_{r,i}=\tau_{s}+M\tau_{r},$$

where $\tau_{r,i}=\tau_{r}$ denotes the reporting time for the $i^{th}$ CR IoT user. While the sensing time is shared by all CR-IoT users, the reporting time is not shared. Therefore, the total time $\tau_{t}$ is dependent on the number of CR-IoT users *M*. As *M* increases, the cooperative sensing performance is improved but the overhead for cooperation increases.
**Algorithm 2** The proposed KLD technique for a CR-IoT network.**Input: $M,N_{s},f_{s},T,\tau_{s},$ and $\tau_{r}$****Output: Calculate the probability of a false alarm $p_{f,FC}^{KLD},$ the probability of detection $p_{d,FC}^{KLD},$ and the sum rate $R^{KLD}$**1:Initialize $N_{s},M$  2:**for***i* from *M* **do**3: Calculate: $e_{i}=\sum_{n=1}^{N_{s}}{\left|z_{i}\left(\frac{n}{f_{s}}\right)\right|}^{2}$  4: Set: $e_{i}∼\left\{\begin{array}{l} ℵ\left(\mu_{0,i}\left(H_{0}\right),\sigma_{0,i}^{2}\left(H_{0}\right)\right)\\ ℵ\left(\mu_{1,i}\left(H_{1}\right),\sigma_{1,i}^{2}\left(H_{1}\right)\right) \end{array}\right.$  5: Calculate: ${\bar{\mu }}_{0,i}=\frac{\sum_{i=1}^{M}\mu_{0,i}-\mu_{0,i}}{\left(M-1\right)}$ and ${\bar{\mu }}_{1,i}=\frac{\sum_{i=1}^{M}\mu_{1,i}-\mu_{1,i}}{\left(M-1\right)}$ with $\mu_{0,i}$ and $\mu_{1,i}$ in Equation (13)  6: Calculate: ${\bar{\sigma }}_{0,i}^{2}=\frac{\sum_{i=1}^{M}\sigma_{0,i}^{2}-\sigma_{0,i}^{2}}{\left(M-1\right)}$ and ${\bar{\sigma }}_{1,i}^{2}=\frac{\sum_{i=1}^{M}\sigma_{1,i}^{2}-\sigma_{1,i}^{2}}{\left(M-1\right)}$ with $\sigma_{0,i}^{2}$ and $\sigma_{1,i}^{2}$ in Equation (14)  7: Calculate: $KLD\left(i\right)=\frac{1}{2}\left[\log\left(\frac{{\bar{\sigma }}_{0,i}^{2}}{{\bar{\sigma }}_{1,i}^{2}}\right)-1+\left(\frac{{\bar{\sigma }}_{1,i}^{2}}{{\bar{\sigma }}_{0,i}^{2}}\right)+\frac{{\left({\bar{\mu }}_{1,i}-{\bar{\mu }}_{0,i}\right)}^{2}}{{\bar{\sigma }}_{0,i}^{2}}\right]$  8:**end for** 9:**if**$\sum_{i=1}^{M}KLD\left(i\right)<\beta_{KLD}$**then**10: Set: $p_{f,FC}^{KLD}=1$  11:**else**12: Set: $p_{f,FC}^{KLD}=0$  13:**end if** 14:**if**$\sum_{i=1}^{M}KLD\left(i\right)\geq \beta_{KLD}$**then**15: Set: $p_{d,FC}^{KLD}=1$  16:**else**17: Set: $p_{d,FC}^{KLD}=0$  18:**end if** 19:Calculate: $R^{KLD}=\alpha p_{d,FC}^{KLD}R_{PU}+\left(1-\alpha \right)\left(1-p_{f,FC}^{KLD}\right)R_{CR-IoT}$

## 4. Simulation Results and Discussion

In this section, the simulation results and the related discussion are presented. To evaluate the performance of the proposed scheme, numerical evaluations were performed and compared with that of several other schemes using the Monte Carlo method. The simulations have been executed using MATLAB 2016a, and the results are obtained from the average of 30,000–70,000 independent simulation runs. The simulation parameters used are listed in Table 1.

**Figure 3:**
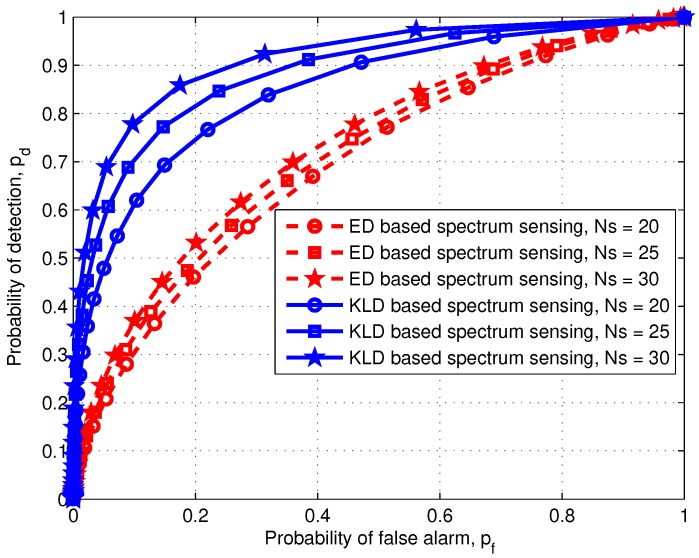
Receiver operating characteristic (ROC) curves at FC of the proposed and conventional schemes.

The sensing performance of the ED and KLD techniques are studied first, and their results are presented in Figure 3. The results were obtained for a fixed SNR (γ=−6 dB) and varying number of samples $\left(N_{s}=20,25,30\right)$. Figure 3 shows the cooperative sensing performance obtained with receiver operating characteristic (ROC) curves for the proposed and the conventional schemes with different numbers of samples. In the conventional scheme, the probability of detection increases with $N_{s}$. For example, the probability of detection $p_{d,FC}^{ED}$ and the probability of false alarm $p_{f,FC}^{ED}$ are 0.52 and 0.20, respectively, when $N_{s}=30$. For the same false alarm probability, the detection probability is lower when $N_{s}=25$ and $N_{s}=20$. In the proposed scheme, the sensing performance is enhanced owing to the KLD technique. For example, the probability of detection $p_{d,FC}^{KLD}$ and probability of false alarm $p_{f,FC}^{KLD}$ are 0.89 and 0.20, respectively. By comparing both the ROCs at the FC in the conventional and proposed schemes, it can be shown that the proposed scheme provides a much better detection performance (71.15%) than the conventional scheme. In addition, when the number of samples $N_{s}=30$, then the detection probability is higher than that in cases with a smaller $N_{s}$ ($N_{s}=20or25$). For example, a KLD method requires 20 samples ($N_{s}=20$) to obtain $P_{d,FC}^{KLD}=0.75$ (for a given $P_{f,FC}^{KLD}=0.2$). If an ED method is adopted, we can obtain $P_{d,FC}^{ED}=0.53$ (for a given $P_{f,FC}^{ED}=0.2$) even with 10 more samples than a KLD method ($N_{s}=30$). Therefore, to obtain the same detection probability, an ED method requires much more sample than a KLD method. Hence, Figure 3 shows that the proposed KLD requires less $N_{s}$ than an ED method to achieve the same sensing performance.

In the comparison of the sensing performance at an FC as in Figure 3, it is observed that with $N_{s}=20$, $N_{s}=25$, and $N_{s}=30$, the proposed scheme can detect the primary spectrum with a detection probability of 75%, 81%, and 89%, respectively, while the conventional scheme detects the licensed spectrum with a detection probability of 46%, 50%, and 53%, respectively, as listed in Table 2.

**Figure 4:**
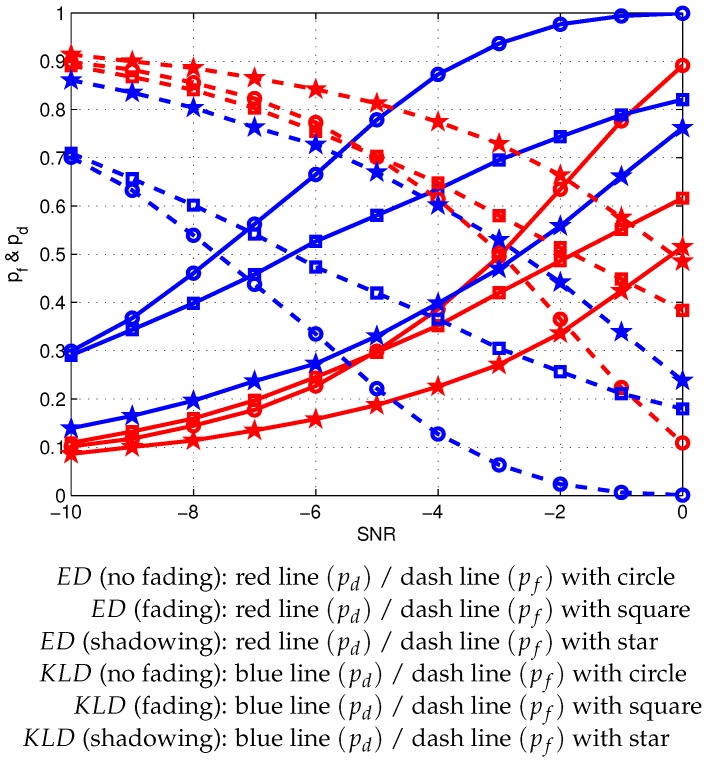
Detection and false alarm probabilities at the FC of the proposed and conventional schemes under various channels with $N_{s}=30$.

As shown in Figure 4, the simulation was performed under conditions wherein the SNR of the PU’s signal at the CR-IoT users varies from −10 dB to 0 dB under fading channels. In a Rayleigh fading channel [41,42,43], the signal amplitude follows a Rayleigh distribution, and the instantaneous SNR of the PU’s signal at the unlicensed CR-IoT then follows an exponential distribution with a PDF that is given as follows:

(22) $$f_{\gamma }\left(\gamma \right)=\frac{1}{\bar{\gamma }}e^{\frac{-\gamma }{\bar{\gamma }}},$$

where $\bar{\gamma }$ is the average SNR value. In a shadowing effect [41,42,43], the signal amplitude follows a log-normal distribution with a PDF that is given as follows:

(23) $$f\left(\gamma_{i}\right)=\frac{1}{\sqrt{2\pi }\sigma_{i}\gamma_{i}}e^{-\frac{\left(\ln\gamma_{i}-\bar{\gamma_{i}}\right)}{2\sigma_{i}^{2}}}.$$

Figure 4 presents the impact of the SNR and the fixed number of samples in the probability detection and the probability of false alarm for the proposed and the conventional sensing scheme. Based on the results, a higher probability of detection can be realized at a higher SNR for both the KLD and the ED processes in channels without fading. However, a lower probability of false alarm can be realized even under a higher SNR for the KLD and the ED schemes under fading channels. It is also shown that the detection performance in the proposed scheme is better compared to that in the conventional scheme under different channels with $N_{s}=30$ number of samples.

To compare the sensing performance at an FC, the probabilities of detection and false alarm are evaluated under various channel models. As shown in Figure 4, the proposed scheme with $\bar{\gamma }=\left[-10:2:0\right]$ dB can detect the spectrum with a detection probability of 12%, 20%, 29%, 40%, 56%, and 78%, respectively, whereas the conventional scheme with $\bar{\gamma }=\left[-10:2:0\right]$ dB detects the licensed spectrum with a detection probability of 9%, 11%, 16%, 22%, 33%, and 51%, respectively, as shown in Table 3.

**Figure 5:**
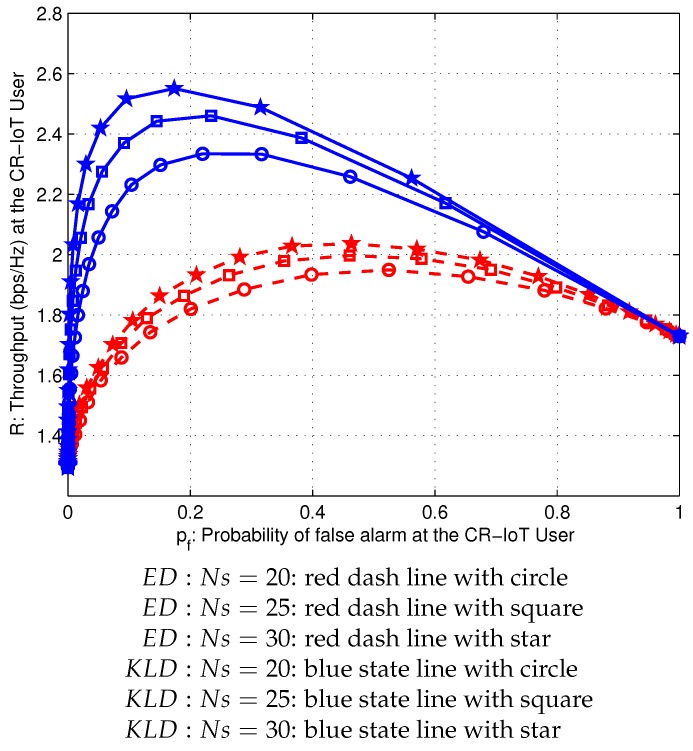
Sum rate curves versus probability of false alarm of CR-IoT user in conventional and proposed schemes when α=0.7.

Figure 5 shows the sum rates for the conventional and proposed schemes depending on the probability of false alarm of a CR-IoT user because the sum rate is a function of the probability of false alarm $\left(p_{f,FC}^{ED}/p_{f,FC}^{KLD}\right)$. The sum rate of the proposed scheme is higher than that of the conventional scheme for the entire range of the probability of false alarm ($p_{f,FC}^{ED}$ and $p_{f,FC}^{KLD}$). Furthermore, the sum rate curve is a quasi-concave function of the probability of false alarm ($p_{f,FC}^{ED}$ and $p_{f,FC}^{KLD}$) for a given primary activity factor α. Therefore, the sum rates of the proposed scheme are 58%, 61%, and 71% over the conventional scheme under the number of samples, $N_{s}=20$, $N_{s}=25$, and $N_{s}=30$, respectively.

To compare the throughput of ED and KLD approaches, the sum rates are evaluated under the different number of samples, e.g., $N_{s}=20$, $N_{s}=25$, and $N_{s}=30$. As shown in Figure 5, the proposed scheme can be obtained as an enhanced sum rate with 2.30 bps/Hz, 2.49 bps/Hz, and 2.58 bps/Hz, respectively, whereas those of the conventional scheme are 1.8 bps/Hz, 1.83 bps/Hz, and 1.98 bps/Hz, respectively, as listed in Table 4.

The results listed in Table 2, Table 3 and Table 4 show that the ED technique cannot realize an acceptable sensing performance with a small number of samples. To achieve a high probability of detection, firstly, the ED technique requires a larger number of samples, i.e., a longer sensing period. Therefore, the proposed scheme realizes a higher probability of detection because the KLD technique performs well even with a small number of samples. The short sensing interval is essential in IoT networks wherein a power saving operation is the most significant issue for a longer life time of the IoT devices.

## 5. Conclusions and Future Works

In this work, the cooperative spectrum sensing performance of the proposed scheme with the KLD technique and conventional scheme with the ED technique has been presented. The proposed scheme can provide a better sensing performance (41.57%) than the conventional spectrum sensing scheme under no fading channel with $N_{s}=30$ samples. The probability of detection in the proposed scheme with SNR=−4 dB is 49%, 25%, and 19% greater than the conventional scheme under no fading, fading, and shadowing fading, respectively. In addition, the proposed scheme provides an enhanced sum rate compared to the conventional scheme. Moreover, the proposed KLD scheme requires less sensing time as compared to the conventional ED scheme while maintaining the sensing performance. For energy-efficient IoT networks, the proposed scheme can achieve a higher sum rate and energy efficiency.

In our future work, we intend to analyze the sensing performance and sum rate using the reporting framework while considering the interference to the PU link. In addition, the dynamic threshold of the KLD technique could be considered under a noise uncertain environment.

## Figures and Tables

**Table 1:** Parameters used in simulations.

Parameter	Value
The total number of CR-IoT users *M*	12
Sampling frequency $f_{s}$	300 kHz
Sensing time slot $\tau_{s}$	300 ms
Reporting time slot $\tau_{r}$	5 ms
PU’s signal x(n)	BPSK
$SNR_{PU}$	10 dB
$SNR_{CR-IoT,i}$	7 dB
Global decision threshold β	3
Number of samples $N_{s}$	[20, 25, 30]
Primary activity factor α	0.7
Average SNR $\bar{\gamma }$	−6 dB

**Table 2:** Sensing performance at the FC for CR-IoT networks with a given false alarm probability ($p_{f,FC}^{ED}=p_{f,FC}^{KLD}=0.2$).

The number of samples in sensing phase	$N_{s}=20$	$N_{s}=25$	$N_{s}=30$
Probability of detection $p_{d,FC}^{ED}$	0.46	0.50	0.53
Probability of detection $p_{d,FC}^{KLD}$	0.75	0.81	0.89

**Table 3:** Sensing performance at the FC of the proposed and conventional schemes under shadowing effects with $N_{s}=30$, $\bar{\gamma }=-10$ dB to 0 dB.

SNR of sensing link ($\bar{\gamma }$)	−10 dB	−8 dB	−6 dB	−4 dB	−2 dB	0 dB
Probability of detection $p_{d,FC}^{ED}$	0.09	0.11	0.16	0.22	0.33	0.51
Probability of false alarm $p_{f,FC}^{ED}$	0.91	0.89	0.84	0.78	0.67	0.49
Probability of detection $p_{d,FC}^{KLD}$	0.12	0.20	0.29	0.40	0.56	0.78
Probability of false alarm $p_{f,FC}^{KLD}$	0.87	0.80	0.72	0.60	0.43	0.23

**Table 4:** Sum rate at an FC for CR-IoT networks with a given false alarm probability ($p_{f,FC}^{ED}=p_{f,FC}^{KLD}=0.2$).

Number of samples in sensing phase	$N_{s}=20$	$N_{s}=25$	$N_{s}=30$
Conventional scheme sum rate $R_{d,FC}^{ED}$	1.8 bps/Hz	1.83 bps/Hz	1.98 bps/Hz
Proposed scheme sum rate $R_{d,FC}^{KLD}$	2.30 bps/Hz	2.49 bps/Hz	2.58 bps/Hz
